# Synthesis with Nitriles: Synthesis of Some New Mercapto-pyridazine, Mercaptopyridazino[1,6-*a*]quinazoline and Thiophene Derivatives

**DOI:** 10.3390/molecules13112750

**Published:** 2008-11-04

**Authors:** Mariam A. Al-Sheikh

**Affiliations:** Chemistry, Girls College of Education, Jeddah, P. O. Box 138016, Jeddah 21323, Kingdom of Saudi Arabia; E-mail: r1425@hotmail.com

**Keywords:** Thiophene, Pyridazine, Pyridazino[1,6-*a*]quinazoline.

## Abstract

2-(1-(4-Bromophenyl)-2-thiocyanatoethylidene)malononitrile (**3**) undergoes azo coupling with diazotized aromatic amines to afford arylhydrazone derivatives, which are readily cyclized to afford the corresponding 3(2*H*)-pyridazinimine derivatives upon reflux in aqueous NaOH. Under similar condition an *o*-cyanoarylhydrazone derivative was cyclized into 6*H*-pyridazino[1,6-*a*]quinazolin-6-imine, which in turn was easily transformed into 6*H*-pyridazino[1,6-*a*]quinazolin-6-one on reflux in ethanolic/HCl. Compound **3** afforded substituted 5-acetylthiophene derivatives upon reflux in AcOH/HCl mixtures.

## Introduction

During the past few decades there has been increasing interest in the synthesis and properties of pyridazines, pyridazinones and pyridopyridazinones. Pyridazines and pyridazino[1,6-*a*]quinazolines show diuretic [1], antihyprertensive [2,3], anticonvulsant, antispasmodic and muscle relaxant activities [3,4]. They inhibit blood platelet aggregation [5] and are active in the treatment of diabetic complications [6]. In addition, these compounds have been tested as cardiac [7] and tuberculostatic agents, as fungicides [4] and as herbicides [8]. Their use as antiasthmatics, analgesics and inflammation inhibitors has also claimed [9]. Recently, the pyridazinone nucleus has been extensively studied in a variety of medicinal agents [10], particularly as an important pharmacophore in the search for drugs acting on the cardiovascular system [11].

**Scheme 1:**
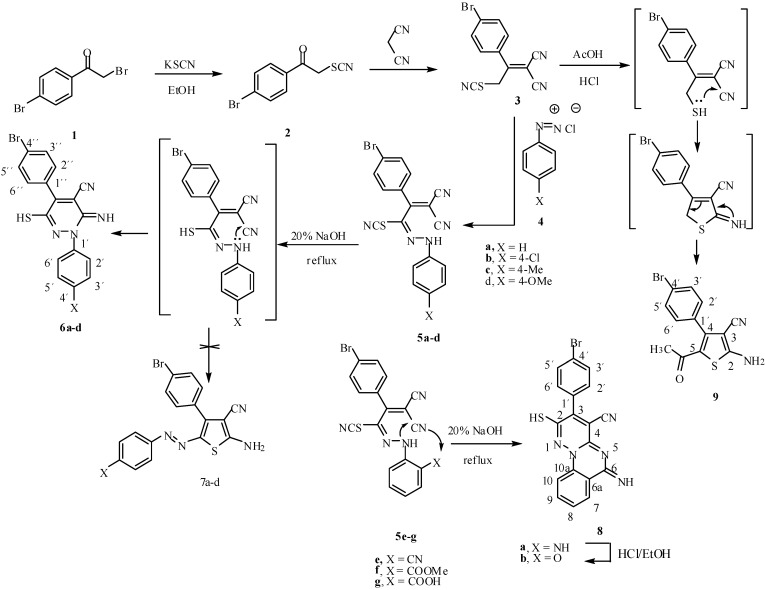
Reactivity of 2-(1-(4-bromophenyl)-2- thiocyanatoethylidene)malononitrile (**3**).

Continuing our interest in synthesis of pyridazines [12,13,14], some new functionally substituted pyridazine and pyridazinoquinazoline derivatives were required. 2-(1-(4-Bromophenyl)-2-thiocyanato-ethylidene)malononitrile (**3**) seemed a good candidate to fulfil this objective via its coupling with the diazotized aromatic amines **4a**-**d** to afford the arylhydrazone derivatives **5a**-**d**, followed by cyclization to the pyridazines **6a**-**d** (Scheme 1), by analogy with previously reported work on related systems [15,16].

## Results and Discussion

It has been found that reaction of compound **1** with potassium thiocyanate in ethanol produced the thiocyanate derivative **2** in 80% yield. Compound **2** condensed with malononitrile in ethanol in the presence of piperidine to afford the Knoevenagel condensation product **3** in 74 % yield. Compound **3** undergoes azo coupling reaction with diazotized aromatic amines to afford the arylhydrazone derivatives **5a**-**g**. Analytical and spectral data of these new arylhydrazone compounds were in complete agreement with the proposed structures. It had been previously reported [17] that similar systems could be cyclized in acidic media, however, in our hands prolonged reflux under such acidic conditions did not produce the desired pyridazine derivatives **6a**-**d**, and we were only able to effect the cyclization of these arylhydrazone derivatives by refluxing in 20% ethanolic sodium hydroxide solution, although the SCN group was simultaneously hydrolysed to a SH group. Thus, compounds **5a**-**d** were cyclized to give the corresponding 3(*2H*)-pyridazinimine derivatives **6a**-**d**, respectively (Scheme 1). This cyclization is assumed to proceed via the hydrazonothiol intermediate, and the other possibility of cyclization to give the thiophene derivatives **7a**-**d** was readily ruled out on the basis of the ^1^H-NMR spectra of the products, which revealed the SH and NH signals at δ = 6.81 and 8.27 ppm, respectively, besides the aromatic protons at 7.52 ppm. In the case of compound **6d**, the ^13^C-NMR and mass spectra were also in agreement with the proposed structure.

The arylhydrazone derivatives **5e** underwent a cyclization reaction under conditions similar to those used for compounds **5a**-**d** to afford the 2-mercapto-6*H*-pyridazino[1,6-*a*]quinazolin-6-imine derivative **8a**, which was assumed to result from a double internal Michael addition of the NH to the neighboring CN group. The IR spectrum of **8a** showed a broad NH absorption band at 3435, 3324 and a CN absorption band at 2224 cm^-1^. Its ^1^H-NMR spectrum revealed two singlets at 6.88 and 8.31 ppm, which were attributed to the SH and NH protons, respectively. The aromatic protons appeared at 7.72 ppm. The elemental analysis of **8a** was in good agreement with the proposed structure.

Compounds **5f** and **5g** underwent a similar cyclization under the same conditions to produce the 2-mercapto-6*H*-pyridazino[1,6-*a*]quinazolin-6-one derivative **8b**, apparently *via* loss of water or methanol, respectively. The IR spectrum of **8b** showed absorption bands at 2214 and 1674 cm^-1^ corresponding to CN and C=O groups, respectively. The ^1^H-NMR spectrum of **8b** revealed only one proton singlet at 6.80 ppm, which was attributed to the SH group, in addition to the aromatic protons at 8.23 ppm. Compound **8b** could be obtained quantitatively from **8a** upon refluxing the latter in ethanolic hydrochloric acid solution. The two products were matched by mixed m.p. and TLC analysis.

On the other hand, thiophene derivative **9** could be obtained in quantitative yield from compound **3** by refluxing in AcOH/HCl mixture for 3 h. The IR spectrum of **9** showed absorption bands at 3402, 2221, 1680 cm^-^1, corresponding to NH_2_, CN and C=O groups, respectively. The ^1^H-NMR spectrum of **9** revealed a singlet at 1.65 ppm (3H) and a singlet at 6.67 ppm (2H), which were attributed to the CH_3_ and NH_2_ groups, respectively, in addition to the four aromatic protons at 7.2 ppm. In the ^13^C-NMR of compound **9**, 11 signals was found; the ones at 28.5, 114.4 and 186.1 ppm were attributed to the CH_3_, CN and CO groups, respectively. A similar result was previously reported [18]. From these data the reaction product could be formulated as the 5-acetyl-2-aminothiophene-3-carbonitrile derivative **9**. The elemental analysis of **9** was in good agreement with the proposed structure (Scheme 1).

## Conclusions

Pyridazinimine derivatives **6**, pyridazino[1,6-*a*]quinazoline **8** and 5-acetylthiophene derivative **9** have been synthesized in good yield *via* 2-thiocyanatoethylidene malononitrile (**3**).

## Experimental

### General

Melting points were measured on a Gallenkamp Electrothermal melting point apparatus and are uncorrected. IR spectra (KBr pellets) were recorded on a Pye Unicam SP 3-300 Spectrophotometer. NMR spectra were recorded in DMSO-d_6_ on Varian Gemini 200/300 MHz NMR spectrometers using tetramethylsilane (TMS) as an internal reference. Mass spectra were registered on a Shimadzu GCMS-QP 1000 Ex mass spectrometer at 70 eV. Elemental analyses were carried out at the Microanalytical Center of Cairo University.

### 1-(4-Bromophenyl)-2-thiocyanatoethanone (**2**)

To a solution of **1** (10 mmol) in EtOH (60 mL) was added KSCN (10 mmol). The reaction mixture was refluxed for 1.5 h. The mixture was then poured on ice-cold water, the solid collected by filtration and recrystallized from EtOH to give yellow crystals, 80% yield, mp. 148-149^o^C; IR (cm^-1^): 2175 (SCN), 1680 (CO); ^1^H-NMR δ: 5.07 (s, 2H, CH_2_), 7.77 (d, 2H, *J* = 8 Hz, Ar-H-3´,5´), 7.94 (d, 2H, *J* = 8 Hz, Ar-H-2´,6´); ^13^C-NMR δ: 41.56 (*C*H_2_), 112.75 (S*C*N), 128.51 (C-4´), 130.54 (C-2´, 6´), 132.03 (C-3´, 5´), 133.35 (C-1´), 190.67 (C-1); MS: 256 (M^+^^79^Br, 14%); 258 (M^+^+2 ^81^Br, 13%); Anal. Calcd. for C_9_H_6_BrNOS (256.12): C, 42.21; H, 2.36; N, 5.47. Found: C, 42.51; H, 2.65; N, 5.70.

### 2-(1-(4-Bromophenyl)-2-thiocyanatoethylidene)malononitrile (**3**)

A mixture of **2** (10 mmol) and malononitrile (10 mmol) was refluxed in EtOH (30 mL) in the presence of piperidine (2 mL) for 3 h, then left to cool at room temperature and the solid product was collected by filtration, washed with EtOH and recrystallized from EtOH to give green crystals, 74 % yield, mp. 258-260^o^C; IR (cm^-1^): 2210 (CN), 2175 (SCN); ^1^H-NMR δ: 5.41 (s, 2H, CH_2_), 7.64-7.72 (m, 4H, Ar-H); MS: 304 (M^+^^79^Br, 56%); 306 (M^+^ +2 ^81^Br, 53%); Anal. Calcd. for C_12_H_6_BrN_3_S (304.17): C, 47.38; H, 1.99; N, 13.81. Found: C, 47.67; H, 2.31; N, 14.15.

### General procedure for preparation of arylhydrazone derivatives **5a**-**g**

To a stirred cold solution of **3** (10 mmol) and sodium acetate (10 g) in EtOH (50 mL) or pyridine (25 mL) was added dropwise over about 30 minutes a cold solution of a diazotized amine (aniline, 4-chloro-, 4-methyl-, 4-methoxyaniline, anthranilic acid, methyl anthranilate or anthranilonitrile, 10 mmol). The stirring was continued for 1h more. The coloured solids were collected by filtration, washed with cold water, and recrystallized from EtOH or 1:1 EtOH/DMF to afford **5a**-**g**, respectively.

*2-[1-(4-Bromophenyl)-2-(phenylhydrazono)-2-thiocyanatoethylidene] malononitrile* (**5a**): Brown crystals (78%); mp. 204-206oC; IR (cm-1): 44308, 3310 (NH), 2214 (CN), 2178 (SCN); 1H-NMR δ: 7.34-7.60 (m, 5H, Ar-H), 7.82 (d, 2H, *J* = 8Hz, Ar-H), 7.95 (d, 2H, *J* = 8 Hz, Ar-H), 9.90 (s, 1H, NH).; MS: 408 (M+, 79Br, 38%); 410 (M++2, 81Br, 35%); Anal. Calcd. for C18H10BrN5S (408.28): C, 52.95; H, 2.47; N, 17.15. Found: C, 53.23; H, 2.71; N, 16.98.

*2-[1-(4-Bromophenyl)-2-[(4-chlorophenyl)hydrazono]-2-thiocyanatoethylidene] malononitrile* (**5b**): Yellow solid (81%); mp. 210-212^o^C; IR (cm^-1^): 4388, 3330 (NH), 2218 (CN), 2175 (SCN); ^1^H-NMR δ: 7.27-7.31 (m, 4H, Ar-H), 7.39-7.43 (m, 4H, Ar-H), 9.95 (bs, 1H, NH); Anal. Calcd. for C_18_H_9_BrClN_5_S (442.72): C, 48.83; H, 2.05; N, 15.82. Found: C, 49.12; H, 2.11; N, 16.17.

*2-[1-(4-Bromophenyl)-2-thiocyanato-2-(p-tolylhydrazono)ethylidene] malononitrile* (**5c**): Yellow solid (74%); mp. 218-219^o^C; IR (cm^-1^): 4395, 3318 (NH), 2221 (CN), 2177 (SCN); ^1^H-NMR δ: 2.27 (s, 3H, CH_3_), 7.10 (d, 2H, *J* = 9Hz, Ar-H), 7.47 (d, 2H, *J* = 9 Hz, Ar-H), 7.64 (d, 2H, *J* = 8 Hz, Ar-H), 7.92 (d, 2H, *J* = 8 Hz, Ar-H), 9.65 (s, 1H, NH); Anal. Calcd. for C_19_H_12_BrN_5_S (422.30): C, 54.04; H, 2.86; N, 16.58. Found: C, 54.34; H, 3.12; N, 16.44.

*2-[1-(4-Bromophenyl)-2-[(4-methoxyphenyl)hydrazono]-2-thiocyanatoethylidene] malononitrile* (**5d**): Yellow solid (74%); mp. 214-216^o^C; IR (cm^-1^): 4395, 3325 (NH), 2224 (CN), 2174 (SCN); ^1^H-NMR δ: 3.81 (s, 3H, OCH_3_), 7.02 (d, 2H, *J* = 9 Hz, Ar-H), 7.44 (d, 2H, *J* = 9 Hz, Ar-H), 7.49 (d, 2H, *J* = 8 Hz, Ar-H), 7.91 (d, 2H, *J* = 8 Hz, Ar-H), 11.05 (s, 1H, NH); Anal. Calcd. for C_19_H_12_BrN_5_OS (438.30): C, 52.07; H, 2.76; N, 15.98. Found: C, 52.37; H, 3.03; N, 16.24.

*2-[1-(4-Bromophenyl)-2-[(2-cyanophenyl)hydrazono]-2-thiocyanatoethylidene] malononitrile* (**5e**): Yellow solid (71%); mp. 205-207^o^C; IR (cm^-1^): 4385, 3330 (NH), 2220, 2212 (CN), 2177 (SCN); ^1^H-NMR δ: 7.49-7.65 (m, 4H, Ar-H), 7.74-8.30 (m, 4H, Ar-H), 10.95 (s, 1H, NH); Anal. Calcd. for C_19_H_9_BrN_6_S (433.29): C, 52.67; H, 2.09; N, 19.40. Found: C, 52.39; H, 2.33; N, 19.24.

*2-[N'-[2-(4-Bromophenyl)-3,3-dicyano-1-thiocyanatoethylidene] hydrazono]benzoic acid methyl ester* (**5f**): Yellow solid (77%); mp. 199-201^o^C; IR (cm^-1^): 3439, 3310 (NH), 2210 (CN), 2175 (SCN), 1668 (CO) cm^-1^; ^1^H-NMR δ: 2.62 (s, 3H, CH_3_), 7.27-7.49 (m, 4H, Ar-H), 7.65-8.30 (m, 4H, Ar-H), 11.10 (s, 1H, NH). MS: 465 (M^+^-1,^79^Br, 31%); 467 (M^+^+1, ^81^Br, 32%); Anal. Calcd. for C_20_H_12_BrN_5_O_2_S (466.31): C, 51.51; H, 2.59; N, 15.02. Found: C, 51.72; H, 2.71; N, 15.30.

*2-[ N'-[2-(4-bromophenyl)-3,3-dicyano-1-thiocyanatoallylidene]hydrazino)benzoic acid* (**5g**): Yellow solid (70%); mp. 225-227^o^C; IR (cm^-1^): 3414, 3335 (NH), 2214 (CN), 2178 (SCN), 1685 (CO); ^1^H-NMR δ: 7.09-7.49 (m, 4H, Ar-H), 7.65-7.98 (m, 4H, Ar-H), 10.66 (s, 1H, NH), 14.03 (s, 1H, COOH); Anal. Calcd. for C_19_H_10_BrN_5_O_2_S (452.29): C, 50.46; H, 2.23; N, 15.48. Found: C, 50.81; H, 2.11; N, 15.61.

### General procedure for preparation of compounds **6a**-**d** and **8a**,**b**

To a solution of each of **5a**-**g** (10 mmol) in EtOH (25 mL) was added 20% aqueous NaOH solution (10 mL). The reaction mixture was refluxed for 2 h, then left to cool. The precipitated solid products formed were collected by filtration, washed with cold water, and recyrstallized from EtOH or 1:1 EtOH/DMF to afford **6a**-**d** and **8a**, **b** respectively.

*5-(4-Bromophenyl)-3-imino-6-mercapto-2-phenyl-2,3-dihydropyridazine-4-carbonitrile* (**6a**)*:* Brown crystals (70%), mp. 233-235^o^C; IR (cm^-1^): 3404, 3318 (NH), 2206 (CN); ^1^H-NMR δ: 6.09 (s, 1H, SH), 7.14-7.42 (m, 2H, Ar-H), 7.52-7.60 (m, 3H, Ar-H), 7.75 (d, 2H, *J* = 8 Hz, Ar-H), 8.17 (d, 2H, *J* = 8 Hz, Ar-H), 8.33 (s, 1H, NH); MS: 383 (M^+^,^79^Br, 19%); 385 (M^+^+2, ^81^Br, 18%); Anal. Calcd. for C_17_H_11_BrN_4_S (383.27): C, 53.27; H, 2.89; N, 14.62. Found: C, 53.33; H, 2.73; N, 14.53.

*5-(4-Bromophenyl)-2-(4-chlorophenyl)-3-imino-6-mercapto-2,3-dihydropyridazine-4-carbonitrile* (**6b**)*:* Brown crystals (64%), mp. 263-265^o^C; IR (cm^-1^): 3400, 3322 (NH), 2209 (CN); ^1^H-NMR δ: 6.45 (s, 1H, SH), 7.54 (d, 2H, *J* = 8 Hz, Ar-H), 7.67 (d, 2H, *J* = 8 Hz, Ar-H), 7.89-8.10 (m, 4H, Ar-H), 8.29 (s, 1H, NH); Anal. Calcd. for C_17_H_10_BrClN_4_S (417.71): C, 48.88; H, 2.41; N, 13.41. Found: C, 48.69; H, 2.54; N, 13.77.

*5-(4-Bromophenyl)-3-imino-6-mercapto-2-p-tolyl-2,3-dihydropyridazine-4-carbonitrile* (**6c**)*:* Brown crystals (64%), mp. 245-247^o^C; IR (cm^-1^): 3390, 3332 (NH), 2212 (CN); ^1^H-NMR δ: 2.34 (s, 3H, CH_3_), 5.87 (s, 1H, SH), 7.24 (d, 2H, *J* = 9 Hz, Ar-H), 7.44 (d, 2H, *J* = 9 Hz, Ar-H), 7.61 (d, 2H, *J* = 9 Hz, Ar-H), 8.26 (d, 2H, *J* = 9 Hz, Ar-H), 8.39 (s, 1H, NH); Anal. Calcd. for C_18_H_13_BrN_4_S (397.29): C, 54.42; H, 3.30; N, 14.10. Found: C, 54.75; H, 3.56; N, 14.34.

*5-(4-bromophenyl)-3-imino-6-mercapto-2-(4-methoxyphenyl)-2,3dihydropyridazine-4-carbonitrile* (**6d**)*:* Brown crystals (69%), mp. 249-251^o^C; IR (cm^-1^): 3395, 3320 (NH), 2208 (CN); ^1^H-NMR δ: 3.65 (s, 3H, OCH_3_), 6.80 (s, 1H, SH), 7.26-7.87 (m, 8H, Ar-H), 8.27 (s, 1H, NH); ^13^C-NMR δ: 55.48 (O*C*H_3_), 89.81 (C-4), 112.43 (CN), 114.83 (C-3´5´), 115.93 (C-4´´), 118.59 (C-2´, 6´), 126.20 (C-2´´,6´´), 129.84 (C-3´´,5´´), 132.08 (C-1´´), 141.88 (C-1´), 148.97 (C-6), 155.42 (C-4´), 159.76 (C-5), 161.46 (C-3); Anal. Calcd. for C_18_H_13_BrN_4_OS (413.29): C, 52.31; H, 3.17; N, 13.56. Found: C, 52.45; H, 3.28; N, 13.84.

*3-(4-Bromophenyl)-6-imino-2-mercapto-6H-pyridazino[1,6-a]quinazoline-4-carbonitrile* (**8a**)*:* Dark brown solid (59%), mp. 314-316^o^C; IR (cm^-1^): 3435 (NH), 2224 (CN); ^1^H-NMR δ: 6.88 (s, 1H, SH), 7.20 (d, 1H, *J* = 8 Hz, Ar-H10), 7.55-7.58 (m, 1H, Ar-H8), 7.68 (d, 1H, *J* = 8 Hz, Ar-H7), 7.77 (d, 2H, *J* = 9 Hz, Ar-H, 2´, 6´), 7.96-7.99 (m, 1H, Ar-H9), 8.24 (d, 2H, *J* = 9 Hz, Ar-H, 3´, 5´), 8.31 (s, 1H, NH); ^13^C-NMR δ: 105.46 (C-4), 116.20 (CN), 121.06 (C-10), 122.08 (C-6a), 125.53 (C-4´), 126.50 (C-7), 126.58 (C-2´, 6´), 128.97 (C-8), 129.98 (C-3´, 5´), 135.64 (C-9), 140.90 (C-1´), 141.22 (C-10a), 150.97 (C-2), 155.80 (C-3), 156.42 (C-4a), 161.95 (C-6); Anal. Calcd. for C_18_H_10_BrN_5_S (408.27): C, 52.95; H, 2.47; N, 17.15. Found: C, 52.79; H, 2.69; N, 17.34.

*3-(4-Bromophenyl)-2-mercapto-6-oxo-6H-pyridazino[1,6-a]quinazoline-4-carbonitrile* (**8b**)*:* Violet crystals (77%), mp. 277-279^o^C; IR (cm^-1^): 2214 (CN), 1674 (C=O); ^1^H-NMR δ: 6.80 (s, 1H, SH), 7.61-7.66 (m, 1H, Ar-H10), 7.81-7.87 (m, 1H, Ar-H8), 7.91-7.99 (m, 1H, Ar-H9), 8.09 (d, 2H, *J* = 8 Hz, Ar-H, 2´,6´), 8.20-8.31 (m, 1H, Ar-H7), 8.86 (d, 2H, *J* = 9 Hz, Ar-H 3´,5´); Anal. Calcd. for C_18_H_9_BrN_4_OS (409.26): C, 52.83; H, 2.22; N, 13.69. Found: C, 53.12; H, 2.44; N, 14.04.

### Transformation of **8a** into **8b** (General procedure)

To a solution of **8a** (50 mmol) in ethanol (25 mL) was added concentrated HCl (5 mL) and the mixture was refluxed for 1 h. After cooling to room temperature, the reaction mixture was diluted with cold water and neutralized with ammonia. The solid formed were collected by filtration and recrystalized from EtOH/DMF (1:1) to afford products identical in all respects (mp, mixed mp and TLC) with **8b**.

### 5-Acetyl-2-amino-4-(4-bromophenyl)thiophene-3-carbonitrile (**9**)

To a solution of **3** (50 mmol) in acetic acid (25 mL) was added concentrated HCl (10 mL) and the mixture was refluxed for 3 h. After cooling to room temperature, the reaction mixture was diluted with cold water and neutralized with ammonia. The solid formed was collected by filtration and recrystalized from EtOH/DMF (1:1) to afford **9**. Yellow crystals (65%), mp. 237-239^o^C; IR (cm^-1^): 3402 (NH_2_), 2221 (CN), 1680 (C=O); ^1^H-NMR δ: 1.65 (s, 3H, CH_3_), 6.67 (s, 2H, NH_2_), 7.08 (d, 2H, *J* = 9 Hz, Ar-H), 7.39 (d, 2H, *J* = 9 Hz, Ar-H); ^13^C-NMR δ: 28.54 (CH_3_), 88.59 (C-3), 114.47 (C-3´,5´), 114.68 (CN), 118.20 (C-4´), 127.92 (C-2´,6´), 132.21 (C-5), 135.20 (C-1´), 147.45 (C-2), 159.37 (C-4), 186.15 (CO); MS: 321 (M^+^); Anal. Calcd. for C_13_H_9_BrN_2_OS (321.19): C, 48.61; H, 2.82; N, 8.72. Found: C, 48.87; H, 3.12; N, 8.89.
